# The effect of the COVID-19 disruption on the gender gap in students’ performance: a cross-country analysis

**DOI:** 10.1186/s40536-023-00154-y

**Published:** 2023-02-23

**Authors:** Alice Bertoletti, Federico Biagi, Giorgio Di Pietro, Zbigniew Karpiński

**Affiliations:** 1 European Commission - Joint Research Centre (JRC), Edificio EXPO, calle Inca Garcilaso, 3, 41092 Seville, Spain; 2 European Commission - Joint Research Centre (JRC), Building 26A CCR, via Enrico Fermi 2749, 21027 Ispra, Italy

**Keywords:** Gender gap, COVID-19, KHB decomposition, I24, J16

## Abstract

**Background:**

This paper investigates how the COVID-19 school closure has affected the gender gap in grade-8 students' performance and what are the drivers behind this. By analysing four different countries (i.e., the Russian Federation, Slovenia, Uzbekistan and the United Arab Emirates), the paper represents the first study addressing the issue from a comparative perspective.

**Methods:**

The study uses data from the Responses to Educational Disruption Survey (REDS) survey, which comprises international comparable data on how students approached remote learning during the COVID-19 disruption. The extent of the gender gap is estimated by employing an ordered logit model, while the Karlson-Holm-Breen (KHB) decomposition method is used to analyse the different potential channels that could account for the gender gap during COVID-19.

**Results:**

The empirical results reveal that, during the COVID-19 school closure, girls tended to perceive changes in their learnings less favourably than boys, both in terms of improvement in self-perceived learning and self-reported improvement in grades—with odds of a more affirmative response between 20 and 25% lower for girls relative to boys. The main drivers explaining this gender gap are physical activity and psychological distress of students during the COVID-19 disruption, as well as the perceived family climate.

**Conclusions:**

The paper shows systematic gender differences in how students perceived their educational outcomes changed due to the COVID-19 disruption, providing evidence on the factors driving these differences. The findings could be employed to design policy actions aimed at increasing gender equality in education.

## Introduction

Since early 2020, educational systems have quickly changed to respond to the disruption caused by the COVID-19 pandemic. In April 2020, school closures affected around 90% of learners worldwide (UNESCO, 2021). Quite abruptly, students were forced to learn how to follow classes, submit their assignments and interact with their classmates and teachers remotely (Schleicher, 2020), with the use of digital devices and often in limited space and resources shared with other family members. The shift to remote learning meant that many students had to quickly improve their digital skills, so as to be able to keep up with the rest of the class; but it also left many of them feeling isolated from their classmates, lacking the emotional support of their friends and teachers—and sometimes also of their parents who were facing a higher risk of unemployment due to economic slowdown (Hoofman & Secord, 2021). Along with the access to the digital devices and the ability to use them efficiently, additional factors related to students’ sense of social isolation, psychological distress, emotional support, subjective well-being, and relationships with peers, teachers, or family, might have affected their academic performance and grades (Di Pietro et al., 2020; Hammerstein et al., 2021).

There is a large body of evidence suggesting that, following the COVID-19 pandemic, the sudden shift from physical presence at school to on line learning has led to a significant learning loss (see, for instance, Maldonado & De Witte, 2022; Engzell et al., 2021). Moreover, many studies show that its negative impact on educational performance does vary across groups of students (Contini et al., 2022; Haelermans et al., 2022).

This paper explores this heterogeneity in depth by looking at how the COVID-19 disruption has affected the gender gap in educational performance. In this way, our work intends to contribute to the extant literature examining the gender gap in educational outcomes, which has extensively grown during the last decades (see, for instance, Di Prete & Jennings, 2012; Bertrand & Pan, 2013; Fortin et al., 2017). Indeed, the determinants of the gender gap identified by this stream of the literature may help us to shed light on the mechanisms through which COVID-19-related school closure might have affected differently the learning outcomes of boys and girls.

While there are already a number of studies suggesting the existence of a gender gap in education caused by the pandemic, our paper aims at expanding this discussion by exploring the channels driving such disparity. In addition, to the best of the authors’ knowledge, this is the first article on this issue that adopts a cross-country perspective. In particular, our research addresses the following research questions:

1. Did the COVID-19 disruption affect differently the learning outcomes of girls and boys?
2. What are the channels explaining the potential gender gap in the learning outcomes during the COVID-19 disruption?

An ordered logit model and the Karlson-Holm-Breen (KHB) decomposition method (Breen et al., 2013) are used to analyse the different potential channels that could account for the gender gap during COVID-19. The empirical analyses are based on the Responses to Educational Disruption Survey (REDS) Student data. A primary focus of this survey is on how students in lower-secondary education (grade 8) responded—not only in terms of learning or digitals skills, but also in terms of health, psychological distress, a sense of social isolation, or subjective well-being—to the educational circumstances caused by the outbreak of the COVID-19 pandemic. Our empirical work analyses information from all the countries for which the Student REDS survey provides representative data: the Russian Federation, Slovenia, the United Arab Emirates (UAE), and Uzbekistan. Being aware that students’ socio-economic conditions and the education systems may remarkably vary across these countries, the primary aim of the paper is to explore common trends of gender differences in students’ performance during the COVID-19 disruption. Additionally, even if our empirical analysis considers a relatively small number of countries, the paper provides a cross-country perspective that is rarely found in the literature—since most of the existing works are based on national data sources.

The remainder of the paper is organised as follows. Section “Literature review” summarises the relevant literature on the topic, while Sect. “Data” presents the data and the descriptive statistics. Section “Methodology” describes our methodological approach. Then, the results and a final discussion about policy implications are presented in Sects. “Results and discussion” and “Conclusions”, respectively.

## Literature review

### Gender differences in the effect of COVID-19 on students’ performance

No clear consensus emerges from research looking at gender differences in the effect of COVID-19 on students’ achievement. Some studies indicate a greater learning loss among girls. For instance, Ardington et al. (2021) investigate the effects of COVID-19 on the reading performance of grade-2 and -4 students in South Africa. They find that girls’ performance in three reading fluency tasks has fallen behind that of boys. On the other hand, other studies produce the opposite result. Employing data from three metro-Atlanta school districts between grades 4 and 8, Sass and Goldring (2021) find that male students have experienced greater reductions in achievement throughout the pandemic than their female peers. Similarly, using data from two surveys conducted during COVID-19 in Pakistan, Crawfurd et al. (2021) conclude that, while girls have achieved the expected progress in math, this was not the case for boys.

There are also studies concluding that there are no statistically significant differences in the way COVID-19 impacted the learning of boys and girls. Engzell et al. (2021) analyse the impact of COVID-19 on the learning outcomes of primary school students (grades 4 to 7) in the Netherlands. Student performance is measured through a composite score of math, spelling and reading. The authors observe that there were no gender-driven differences during the school closure. This result is consistent with that reported by Haelermans et al. (2022) who also focus on Dutch student performances in math, spelling and reading (in this case each score is analysed separately). Yu (2021) also finds no significant differences in online learning outcomes during the pandemic between male and female higher education students in China. Finally, using Uwezo data covering children aged 6 to 16, Sandefur (2022) shows that in Uganda the gender gap in English reading outcomes and math results has not changed as a result of COVID-19.

Other studies suggest that the pandemic may have induced more disadvantaged families to redirect their scarce resources to give priority to the education of boys over girls (de Paz Nieves et al., 2021), thereby undermining the latter’s educational achievement. Contini et al. (2022) analyse how COVID-19 driven school closure affected math test scores of primary school pupils (grades 2 and 3) in Turin (Italy). While the pandemic led to an overall decline in achievement, the learning loss was especially large among girls whose parents have a low level of education. A similar finding is obtained by Hevia et al. (2022) who use data from two household surveys in Mexico and look at children between the age of 10 and 15. Their results indicate that the pandemic had a particularly detrimental effect on the math performance of girls from low socio-economic status. This is consistent with the hypothesis that in households characterised by disadvantaged backgrounds, girls are more likely to have spent less time studying at home than boys - compared to what happened in households with more advantaged backgrounds (Akmal et al., 2020). In the same vein, there are also studies reporting that, due to COVID-19, female students are more likely to have taken up household responsibilities, with potential negative implications for their learning. For instance, using data from a sample of high school students in Ecuador during the COVID-19 lockdown, Asanov et al. (2020) find that females were more likely to be involved in household tasks (e.g., meal preparation, cleaning, laundry, looking after younger siblings) than males.

Finally, there is some evidence showing that the gender impact of COVID-19 on students’ achievement differs across subjects. Employing longitudinal data, Wolf et al. (2021) conclude that the pandemic had a similar effect on the math performance of Ghanian boys and girls aged 9–11, while the effects on literary scores were different between boys and girls (with girls scoring higher than boys). Borgonovi and Ferrara (2022), using data from a sample of Italian secondary school students, find that the pandemic had a larger negative effect on the math achievement of boys relative to girls, whereas the opposite holds for reading scores.

### Potential channels leading to gender differences

Six different channels can be put forward in an attempt to explain why the COVID-19 pandemic may have had differential effects on the learning performance of boys and girls.

#### Psychological distress

There is a large body of evidence showing that students have experienced rising stress levels as a result of the pandemic and its restrictions (see, for instance, Mushquash & Grassia, 2021). The sudden switch from face-to-face to online teaching, social distancing and fears of contagion have all had a detrimental effect on students’ well-being. This may in turn have negatively affected their academic performance, given the close association between psychological well-being and educational outcomes among adolescents (see, for instance, Dalsgaard et al., 2020). Interestingly, some evidence points to cross-gender differences in the effect of COVID-19 on students’ well-being. For instance, Prowse et al. (2021), using data from a survey conducted during the pandemic among students from Canada, find that girls were more likely to report social isolation as being difficult or very difficult compared to males. Similarly, the former were also more likely to respond that COVID-19 negatively impacted their social relationships very much or an extreme amount compared to the latter.

#### Family climate

The COVID-19 lockdown has forced students from practically all over the world to study from home. However, schools are considered a protective and nurturing space by a large number of children, especially the most vulnerable ones. These students may perceive the home learning environment as unsafe, disruptive, and less conducive to learning. There is also evidence showing that the pandemic and the economic stress that followed have led to increased tension and domestic violence within households (Usher et al., 2020). Not only could this situation have negatively affected student performance, but it also may be associated with gender differences. For instance, Baldry (2003) finds that in Italy female children are more likely to have been exposed to domestic violence than male children. However, the opposite conclusion is reached by Hamby et al. (2011).

#### Household resources for remote education

Gender norms and expectations could have influenced how parents reacted to the challenges of COVID-19, resulting in different learning opportunities for boys and girls (UNESCO, 2021). As outlined earlier, especially among more disadvantaged families, these exceptional circumstances could have induced parents to prioritise the education of boys relative to that of girls. This means that not only were the former given the possibility to invest more time in learning than the latter, but they were also more likely to have access to the technology required to study online as well as to a quiet place to study (MIET AFRICA, 2021).

#### Physical activity and fitness

Physical activity is known to have a positive effect on learning (see, for instance, Donnelly et al., 2016). Unfortunately, its amount declined amid the coronavirus crisis, with potentially negative effects on well-being and learning. Yomoda & Kurita (2021) find that in many countries the pandemic led to a drop in physical activity among children. Several studies also point out that the impact of COVID-19 on physical activity did vary across gender. Dallolio et al. (2022) and Sekulic et al. (2020) report that, while boys saw their physical activity levels decline during the pandemic, this did not occur with girls. In contrast to this, Karuc et al. (2020) show that moderate-to-vigorous physical activity levels decreased more in females than in males during COVID-19. Similarly, Moore et al. (2020) find that following the pandemic, girls aged 5 to 11 were less likely to participate in sufficient physical activity than boys of the same age.

#### Support from teachers and family

Both parents and teachers played a critical role in supporting children’s home learning during the lockdown. However, several papers show that there have been differences in the extent of (perceived) support between girls and boys. For instance, Korlat et al. (2021), using data from Austrian secondary school students, find higher levels of perceived teacher support among female students than male students. The former were more likely to reach out to their teachers and develop better relationships with them compared to the latter. In a similar vein, Bol (2020) shows that Dutch parents felt more capable of supporting the learning of their daughters relative to their sons. On the other hand, Ribeiro et al. (2021) argue that in Portugal parents were more involved in the learning of their sons compared to their daughters. Anders et al. (2021) show that during school closures in the UK, boys were more likely to receive private tuitions than girls.

#### ICT skills pre-COVID

Finally, the differential gender effect of the pandemic on student learning could also be explained by the divergent levels of digital skills across boys and girls before COVID-19. Amaro et al. (2020) show a considerable gender ICT skills gap in favour of boys among adolescent students in 7 out of 8 sub-Saharan African countries. This finding seems to be in line with that of Greier et al. (2022), who find that in Austria female university students reported greater difficulties than their male counterparts regarding the use of new software learning programs that were adopted following the COVID-19 lockdown. In the International Computer and Information Literacy Study (ICILS), a considerable gender gap in favour of girls is found on a task-based, standardised test of computer and information literacy. At the same time, girls have a lower confidence in their own digital skills than boys, especially in the area of specialised applications (Fraillon et al., 2020; Gebhardt et al., 2019).

## Data

This paper uses unique data from the REDS Student Database, which comprises international comparable data on how students approached remote learning during the COVID-19 disruption. In this survey, grade-8 students are asked to reflect on their life during the lockdown and examine how their personal, family and academic circumstances have changed compared to the pre-lockdown period.^1^ In many countries, grade 8 coincides with the end of compulsory education and the first significant educational transition that students face. It is for this reason that major large-scale educational surveys target students in grade 8. The administration period of the survey lasted around one month and took place between December 2020 and July 2021, depending on the specific country (see Meinck et al., 2022, for additional information on the reference and administration periods). The empirical analyses are based on data from the Russian Federation, Slovenia, the UAE and Uzbekistan, which represent the total set of countries for which the Student REDS database provides representative data.^2^ As shown by the IEA and UNESCO's report on the REDS data (Meinck et al., 2022), these countries and their educational systems responded differently and were differently prepared to this global challenge. For example, the school closure duration due to COVID-19 varied from 3 weeks in the Russian Federation to 9 months in the UAE.^3^

Two complementary indicators of the change in learning performance due to the COVID-19 school closure can be found in REDS data. The first captures how students self-evaluated their academic performance during the lockdown period relative to the period before the lockdown (i.e., improvement in perceived learning). The variable is built from question IS1G14B of the REDS Student survey: “To what extent do you agree or disagree with the following statement about your learning during the COVID-19 disruption?—I learned more studying at home than when attending regular lessons at school”. The indicator is measured on a Likert scale, with value equal to 1 for "strongly disagree", 2 for "disagree", 3 for "agree" and 4 for "strongly agree". The mean value is 2.17 over the 11,638 observations available.

Our second indicator looks at differences in students’ grades between the lockdown period and the period before the lockdown (i.e., improvement in grades). The variable is built from question IS1G14I of the REDS Student survey: “To what extent do you agree or disagree with the following statements about your learning during the COVID-19 disruption?—I got higher grades than before the COVID-19 disruption”. As for the previous variable, the indicator is measured on a Likert scale, with value equal to 1 for "strongly disagree", 2 for "disagree", 3 for "agree" and 4 for "strongly agree". The mean value is 2.592 over the 11,606 observations available.

The two indicators differ with respect to their scope. Improvement in grades refers to just one specific aspect of the educational process: grades given to students by their teachers. The other indicator, in turn, is broader in scope: it covers students’ learning, not restricted to grades or test scores. What is more, the learning that the question asks about might not refer solely to the learning of the material taught in particular classes. For example, some students could have found it easier to be active during classes conducted online than during classes taught in person. All in all, improvement in perceived learning can be seen as including improvement in grades in its scope. Further remarks on the validity of both measures are presented in Sect. “Conclusions”.

**Fig. 1:**
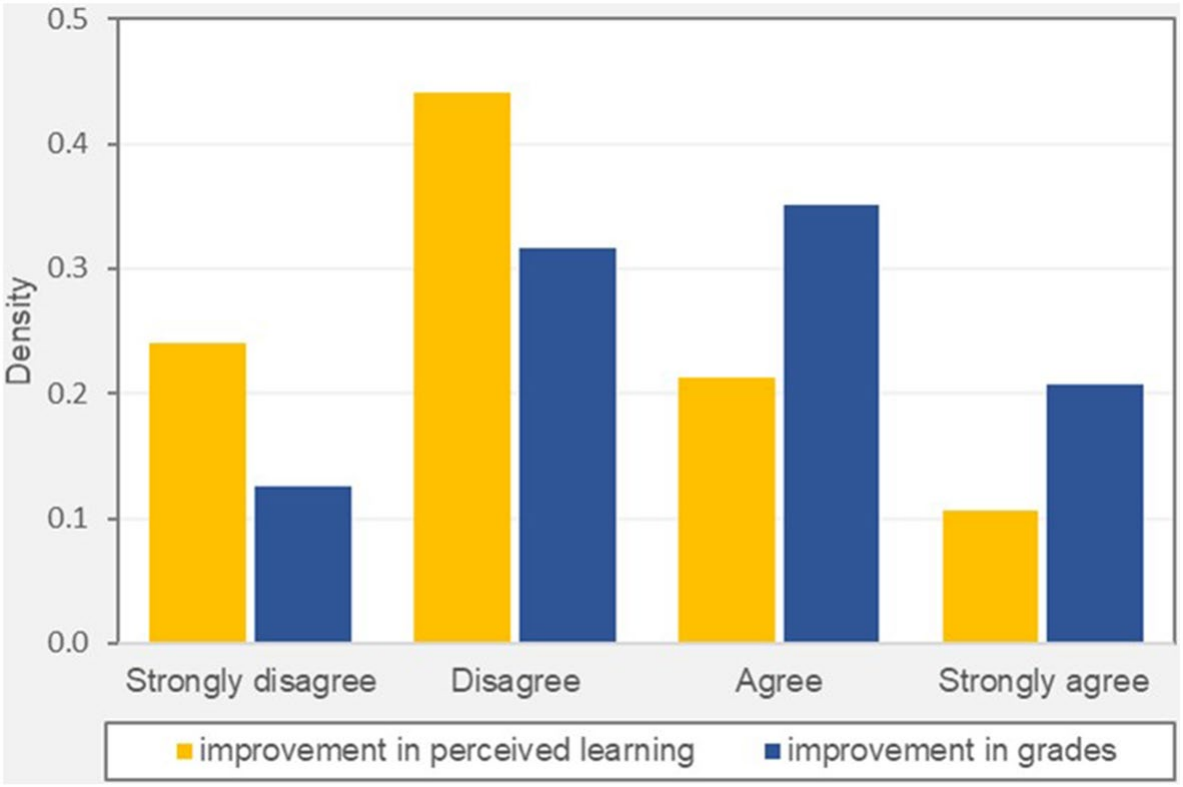
Improvement in perceived learning and grades—distributions compared

Figure 1 shows the differences in the distribution of the two outcome variables. The data indicate that around half of the respondents reported an improvement in their grades during the lockdown compared to the pre-lockdown period (combining “agree” and “strongly agree” responses). On the other hand, around two thirds of students declared to have learned less during physical school closure, compared to the pre-COVID-19 period (combining “disagree” and “strongly disagree” responses). The difference between the two variables may suggest the existence of grade inflation during the COVID-19 emergency period (see Karadag, 2021). While Sanchez and Moore (2022) suggest that grade inflation increased faster for girls than boys between 2020 and 2021, they also observe that the gender gap in favour of girls in grade inflation already existed in the pre-COVID-19 period and its magnitude was higher in 2016 than in 2021.

The REDS database also provides information on students' gender, which represents the variable of primary interest of this study, as well as additional students’ characteristics. Among these, there are students’ age and socioeconomic status, the language spoken at home and if parents have higher educational attainment^4^ (see Table 1 for details).

**Table 1:** Descriptive statistics: variable of interest and controls

Variables' names	Description	Survey REDS code	N. observations	Possible values	Mean
female	Student's gender	IS1G32	11,957	Binary: 1 (female); 0 (male)	0.504
ses	Student's socioeconomic status. The value is provided by IEA and is based on information about the number of books at home, parents' educational level, spoken language at home, parents' employment and type of job, material goods and commodities	SES_irt	11,825	Continuous: overall international average of 50; standard deviation of 10 points on the scale	50.194 (0.282)
age	Student's age	ASDAGE	11,965	Continuous, ranging between 10 and 18	14.476 (0.011)
lang_istr	The variable indicates if the language spoken at home by the student is the same of the language of instruction	IS1G34	11,743	Binary: 1(as the language of instruction); 0 (different from the language of instruction)	0.907
he_parents	The variable indicates if at least one parent has obtained a higher education degree (ISCED level 6, 7 or 8)	IS1G38	11,633	Binary: 1(at least one parent with higher education); 0 (both parents without higher education)	0.463

Means refer to the total sample with survey weights and 75 Jackknife replications. Jackknife standard error in parenthesis (only for continuous variables)

In addition, interestingly, the REDS database can be exploited to investigate the different channels potentially related to gender differences in learning outcomes during COVID-19 (see Sect. “Potential channels leading to gender differences”): psychological distress (PSY), support from teachers and family (SUP), family climate (FAM), physical activity and fitness (PHY), household resources for remote education (RES), ICT skills pre-COVID-19 (ICT).^5^ The six channels are captured by a number of variables whose description is reported in Table 2. More specifically, psychological distress (PSY) is proxied by a set of categorical variables that indicate the degree of students’ anxiety about the changes introduced by the pandemic in their schools and the extent to which they were worried about their present and future education. Thus, the variable focuses on psychological distress related to learning without explicitly including general psychological attitudes of students (which would be difficult to measure in a comprehensive way). Relevant data also shown in Table 2 are consistent with the literature, with students reporting, in general, to have been psychologically distressed during the lockdown, especially in terms of worry for their future education. Regarding the support from teachers and family (SUP), REDS provides information on whether a parent or, more generally, someone was available to help the students with the schoolwork. Support from teachers is, instead, measured through a variable aggregating different questions on teachers' support and availability perceived by students. Looking at the average values in Table 2, around 70% of the students had someone helping them with their schoolwork; however, only 33% of the respondents indicated that the support came from their parents. On the other hand, students were, in general, satisfied with the support received from the teachers. Family climate channel (FAM) is measured, instead, by two categorical variables that indicate the degree of home safety perceived by the students and whether they were happy to be at home during the school closure. On average, students reported feeling safe and happy most of the time. Moreover, physical activity and fitness (PHY) have been modelled by three variables that indicate if students exercised more than usual, whether they increased their outdoor activities during the lockdown and if they felt fit and healthy. On average, the descriptive statistics do not show a relevant change in the physical habits of the students, who generally felt fit and healthy. Regarding household resources for remote education (RES), REDS data allows capturing if students had a personal digital device for studying, a well-working internet connection and a quiet space to study. While proper internet connections and study places were available for most of the students, only around half of the overall sample had their own personal device. Finally, ICT skills pre-COVID-19 (ICT) are measured by the number of tasks that students reported to be able to complete before the pandemic regarding both general digital competencies and the ones specific for remote learning. Descriptive statistics show that students lacked school-specific digital skills, being able to complete, on average, 2 tasks out of 4 (see Table 2).

**Table 2:** Channel variables: definition and descriptive statistics

Channels	Variables' names	Description	Survey REDS code	Possible values	Mean
Psychological distress (PSY)	anxiety	The variable indicates if the student felt anxious about the changes in his/her schooling	IS1G24A	Categorical: 1 (strongly disagree); 2 (disagree); 3 (agree); 4 (strongly agree)	2.895
	worry_learn	The variable indicates if the student was worried about how the disruption affected his/her learning	IS1G24D	Categorical: 1 (strongly disagree); 2 (disagree); 3 (agree); 4 (strongly agree)	3.023
	worry_future	The variable indicates if the student was worried about how this disruption will affect his/her future education	IS1G24E	Categorical: 1 (strongly disagree); 2 (disagree); 3 (agree); 4 (strongly agree)	3.050
Support from teachers and family (SUP)	gen_sup	The variable indicates if there was someone available who could help the student with his/her schoolwork during the COVID-19 disruption. The information, available from IS1G15D, is rescaled as described in “possible values”	IS1G15D	Binary: 1(often or always available); 0 (no or rarely available)	0.679
	parent_sup	The variable indicates if student's parent(s) or guardian(s) was/were available and could help him/her with schoolwork during the COVID-19 disruption. The information, available from IS1G15A, is rescaled as described in “possible values”	IS1G15A	Binary: 1(often or always available); 0 (no or rarely available)	0.330
	teach_sup	The variable is the aggregation of seven questions about the support and the availability of teachers reported by students (availability to help, availability and effort to communicate, quality of feedback, learning motivation, adaption of schoolwork to individual needs). The variable is the mean of the value of the seven variables, being each variable measured in 1–4 Likert scale, with 1 indicating “strongly disagree”, 2 “disagree”, 3 “agree”, and 4 “strongly agree”, for this reason it is treated as continuous	IS1G21A—IS1G21H	Continuous: between 1 and 4	2.947
Family climate (FAM)	safe_home	The variable indicates if the student felt safer at home than she/he usually feels at school	IS1G17B	Categorical: 1 (never or hardly ever); 2 (sometimes); 3 (most of the time); 4 (always)	2.847
	happy_home	The variable indicates if the student was happy to be at home	IS1G17F	Categorical: 1 (never or hardly ever); 2 (sometimes); 3 (most of the time); 4 (always)	2.708
Physical activity and fitness (PHY)	exercise	The variable indicates if the student exercised (including walking) more than usual, during the COVID-19 disruption	IS1G25A	Categorical: 1 (strongly disagree); 2 (disagree); 3 (agree); 4 (strongly agree)	2.743
	outdoor	The variable indicates if the student was able to do more than his/her usual outside-of-school activities (e.g., scouts, guides, training for sports), during the COVID-19 disruption	IS1G25B	Categorical: 1 (strongly disagree); 2 (disagree); 3 (agree); 4 (strongly agree)	2.755
	fit	The variable indicates if the student felt fit and healthy during the COVID-19 disruption	IS1G25C	Categorical: 1 (strongly disagree); 2 (disagree); 3 (agree); 4 (strongly agree)	3.056
Household resources for remote education (RES)	private_pc	The variable indicates if the student had a computer or tablet device used only by him/her, during the COVID-19 disruption	IS1G05A	Binary: 1(yes, it worked well); 0 (no or not working well)	0.524
	internet	The variable indicates if the student had access to the internet at home, during the COVID-19 disruption	IS1G03	Binary: 1(yes, it worked well); 0 (no or not working well)	0.855
	quiet_space	The variable indicates if the student had a quiet space to study with a desk and chair, during the COVID-19 disruption	IS1G17A	Binary: 1(most of the time); 0 (otherwise)	0.828
ICT skills pre-Covid (ICT)	ict_gen	The variable indicates how many general ICT tasks the student reported to already knew how to perform before COVID-19, among: connect a device to the internet, send and receive email, maintain devices, fix problems when the device is not working properly. The variable is built on the information of four single questions, to which the student could respond “I already knew how to do this before the pandemic”, I learned how to do this during the pandemic”, and “I still do not know how to do this”	IS1G07A, IS1G07B, IS1G07H, IS1G07I	Numerical: between 0 and 4 (tasks)	2.906
	ict_school	The variable indicates how many school-related ICT tasks the student reported to already knew how to perform before COVID-19, among: log into my school portal, manage settings on a device to access school software, send photos, videos or audio of my learning, use applications (e.g., Seesaw and Google classrooms) to communicate with my teacher. The variable is built on the information of four single questions, to which the student could respond “I already knew how to do this before the pandemic”, I learned how to do this during the pandemic”, and “I still do not know how to do this”	IS1G07C, IS1G07E, IS1G07F, IS1G07G	Numerical: between 0 and 4 (tasks)	2.127

Mean values refer to the total sample with survey weights and 75 Jackknife replications

## Methodology

An ordered logistic model is employed to assess the existence of a gender gap in the change (i.e., improvement) of perceived learning and grades during the COVID-19 disruption. The ordered logistic regression is an extension of the standard logistic regression for binary variables to dependent variables with more than two response categories that are ordered in a non-arbitrary way. The focus of the analysis is on modelling the odds of giving a more affirmative (e.g., responding “agree” or “strongly agree”), rather than a more negative response (e.g., responding “disagree” or “strongly disagree”) to the questions about improvement in perceived performance or improvement in grades. The results of the analysis are interpreted in terms of odds ratios, i.e., in terms of how the odds of a more affirmative vs more negative response change depending on the values of the independent variables. In this approach, the effects of the independent variables are multiplicative: if the effect of a given independent variable is associated with an odds ratio of *k*, it means that a unit increase in the value of the variable multiples the odds of a more affirmative response by a factor of *k*. To give a more concrete example, if gender is coded 1 for girls and 0 for boys and the effect associated with gender is, say, 0.8, it means that the odds of a more affirmative response for girls are 0.8, or 80%, of the corresponding odds for boys. This is to say that the odds of a more affirmative response are 20% lower for girls than for boys. Values of the odds ratios between 0 and 1 indicate a negative effect of a variable, while values greater than 1 indicate a positive effect. For example, if the effect of gender were 1.2 on the odds ratio scale, it would mean that the odds for girls are 1.2 times the odds for boys, or that the odds for girls are 20% higher than the odds for boys.

Firstly, the baseline model considers the effect of gender and a few other individual characteristics (students’ age and socioeconomic status, the language spoken at home and if parents have higher educational attainment) on the odds ratio for the two dependent variables capturing changes in learning during the pandemic.

The second part of the analysis is concerned with exploring the role of some channels potentially accounting for the observed differences in educational performance between boys and girls. These additional variables are added to the ordered logistic regression, in order to test their individual and collective effect and whether the gender gap in the two dependent variables is confirmed when they are included. Then, a KHB decomposition for ordered logistic models is applied (Breen et al., 2013). The main advantage of this method is that it provides an unbiased decomposition of the total effect of a variable of interest in a logistic regression model into the direct and indirect (or mediated) part (see Kohler et al., 2011). Another central characteristic of the KHB is that, in addition to the mediator variables, this approach allows the inclusion of control (or context) variables in the model (Karlson & Holm, 2011). More specifically, the KHB decomposition involves a comparison of two logistic models: a full model, which provides an estimate of the direct effect of the variable of interest (i.e., gender in our case) and a reduced model, which estimates the total effect of the variable, with the reduced model nested in the full model. With ${\beta }_{R}$ representing the coefficient of the variable of interest (i.e., gender) in the reduced model, and ${\beta }_{F}$ being the coefficient in the full model,^6^ the percentage change in the coefficients attributable to confounding (mediation) can be expressed as (see Karlson et al., 2012, for a derivation):

$$100\times \frac{{\beta }_{R}-{\beta }_{F}}{{\beta }_{R}}$$

Note that this quantity can be negative, which would mean that the mediating variables and the variable of interest have effects opposite in sign on the dependent variable.

## Results and discussion

### Gender gap in students’ learning during COVID-19

Table 3 shows the results of ordered logit regressions on improvement in perceived learning and grades. The estimated coefficients are reported as odds ratio and, thus, values lower than 1 indicate a negative effect of the predictor on the dependent variables. Columns 1 and 3 report the estimates for the baseline model. The results highlight a consistent gender gap (values < 1) both in the case of improvement in perceived learning and improvement in grades: the odds of a more affirmative response are 25% lower for girls relative to boys (i.e., $100\times \left(1-0.755\right)\approx 25\mathrm{\%}$) for improvement in perceived learning, and 22% lower ($100\times \left(1-0.783\right)\approx 22\mathrm{\%}$) for improvement in grades.

**Table 3:** Ordered logit on improvement in perceived learning and in grades, odds ratios estimates

Variables	(1)	(2)	(3)	(4)
	IPL	IPL	IG	IG
Female	0.755***	0.901*	0.783***	0.768***
	(0.048)	(0.053)	(0.041)	(0.043)
parent_sup		0.962		0.978
		(0.067)		(0.064)
gen_sup		0.878*		1.133*
		(0.060)		(0.075)
teach_sup		1.295***		0.949
		(0.073)		(0.062)
anxiety		0.964		1.153**
		(0.041)		(0.077)
worry_learn		0.830***		1.115
		(0.038)		(0.0923)
worry_future		0.968		1.194**
		(0.047)		(0.088)
safe_home		1.176***		1.020
		(0.034)		(0.030)
happy_home		1.401***		0.960
		(0.044)		(0.029)
outdoor		1.089**		1.030
		(0.035)		(0.0404)
exercise		1.168***		1.056
		(0.059)		(0.0443)
fit		1.153***		1.085*
		(0.052)		(0.051)
private_pc		1.303***		1.023
		(0.098)		(0.0459)
internet		1.059		0.847*
		(0.098)		(0.069)
quiet_space		0.789***		0.945
		(0.062)		(0.072)
ict_gen		0.935**		1.002
		(0.025)		(0.027)
ict_school		1.026		0.980
		(0.028)		(0.028)
Controls	Yes	Yes	Yes	Yes
Country FE	Yes	Yes	Yes	Yes
cut1	1.444	5.577*	1.444	0.200**
	(1.230)	(5.226)	(1.230)	(0.154)
cut2	12.849***	60.862***	12.849***	1.329
	(10.848)	(56.262)	(10.848)	(1.022)
cut3	51.415***	265.960***	51.415***	6.474**
	(43.787)	(246.340)	(43.787)	(4.969)
Pseudo R2	0.010	0.060	0.010	0.022
Observations	11,372	10,348	11,372	10,348

***Indicates significance at the 1% level, ** at the 5% level and * at the 10% level. Coefficients are reported in odds ratios. Jackknife standard errors in parentheses. IPL indicates the dependent variable improvement in perceived learning, while IG the improvement in grades. All models include country fixed effects (FE) and the following controls: age, ses, he_parents and lang_istr. Control variables estimates are not included for reasons of space and are available upon request. Results are adjusted for survey setting with 75 Jackknife replications and 43 number of strata. Pseudo R2 is not provided for survey setting. The values of Pseudo R2 are taken from simple ordered logistic models, without survey parameters

Table 7 in the Appendix reports the estimates of the baseline ordered logit on improvement in perceived learning and grades separately for each country. The results confirm the existence of a gender gap in both the dependent variables for all the four countries analysed. The estimated coefficients vary slightly across countries, but without statistically significant differences.^7^

Columns 2 and 4 add the channel variables to the baseline model. The results show that the gender gap in improvement in perceived learning and grades holds even when these variables are included. However, two effects are noticeable. First, in the case of IPL (Column 2), the significance of the gender coefficient drops to 10%. Second, for the improvement in perceived learning, the size of the effect is remarkably reduced, moving from 25 to 10%. All this suggests that the channels, considered together, can account for a relevant part of the gender gap in perceived learning. On the other hand, their joint and individual effect seems to be rather weak for the improvement in grades (the gender gap is almost unchanged). This result is confirmed by the estimated coefficients associated with the channel variables, which have higher statistical significance in Column 2 than in Column 4 and, more importantly, by the Pseudo R squared that is much higher in Column 2 than in Column 4. More generally, the estimates for the coefficients of the channel variables reveal heterogeneous results between Column 2 and Column 4 (the detailed discussion of the effects of the channel variables on students’ outcomes is left to Sect. “Channels expanding the gender gap”).

Finally, Tables 8 and 9 in the Appendix replicate the estimates in Columns 2 and 4 by country. The lack of statistical significance of the gender coefficient for some countries (i.e., the Russian Federation and the UAE for improvement in perceived learning; Slovenia and Uzbekistan for improvement in grades) confirms the existence of a relevant effect produced by the channel variables.

### Channels expanding the gender gap

To explore the role of the channel variables in explaining the gender gap, first, it may be useful to look at the differences in the average values of these indicators between girls and boys. Table 4 reports the means for all the indicators used to measure the six potential channels, as described in Table 2. Females reported a lower level of teacher support, with statistically significant differences in the Russian Federation and Uzbekistan, while parental and general family support does not show a significant gender gap when the overall sample is considered. Girls also exhibited significantly higher levels of psychological distress in all three indicators. The results are consistent across countries, except Uzbekistan, where no statistical difference between girls and boys has been found in terms of worry about their present and future education. Furthermore, the results show a clear difference in the perception of family climate across gender. Boys were, indeed, feeling safer and happier at home compared to girls. However, the result is not confirmed in Slovenia, where female students reported a higher level of home safety. This difference may be related to heterogeneities in cultural values and gender norms across countries. Indeed, based on the 2019 Gender Inequality Index^8^ (GII), Slovenia was the country with the lowest level of gender-based disadvantage among the four countries analysed^9^ (UNDP, 2022). Compared to girls, boys were also more likely to feel healthy and to have increased their outdoor and physical activities compared to the period before the lockdown. The size of the gap is large and consistent across countries. This result is in line with the work of Sajwani et al. (2022) reporting that in the UAE boys tend to do more moderate to vigorous physical activity than girls and suggesting that the pandemic could have exacerbated this gap. Moreover, in terms of household resources, girls were less likely to have a personal laptop (except in Uzbekistan) but, in Slovenia and Uzbekistan, they reported having a quiet space to study more frequently than boys. Finally, before the COVID-19 disruption, girls had levels of ICT skills remarkably lower than male students. The results are consistent for all countries and hold both for general and school-related ICT skills.^10^

**Table 4:** Channel variables mean values—differences between boys and girls

Variable		Total Sample			Russian Federation			Slovenia			United Arab Emirates			Uzbekistan		
		Boys	Girls	p-value	Boys	Girls	p-value	Boys	Girls	p-value	Boys	Girls	p-value	Boys	Girls	p-value
SUP	parent_sup	0.333	0.327	ns	0.322	0.296	ns	0.379	0.406	ns	0.350	0.348	ns	0.375	0.401	ns
	gen_sup	0.673	0.684	ns	0.671	0.681	ns	0.622	0.696	***	0.563	0.629	***	0.675	0.684	ns
	teach_sup	3.058	3.029	*	2.949	2.885	***	2.940	2.947	ns	3.197	3.186	ns	3.300	3.380	***
PSY	anxiety	2.712	2.791	***	2.603	2.744	***	2.603	2.746	***	2.837	2.962	***	3.010	2.914	**
	worry_learn	2.844	2.974	***	2.742	2.926	***	2.605	2.893	***	2.956	3.073	***	3.124	3.098	ns
	worry_future	2.771	2.913	***	2.649	2.838	***	2.525	2.776	***	2.911	3.033	***	3.095	3.108	ns
FAM	safe_home	2.896	2.798	***	2.942	2.837	*	2.642	2.749	**	2.767	2.730	ns	2.790	2.670	**
	happy_home	2.796	2.622	**	2.999	2.824	***	2.942	2.879	ns	2.782	2.609	***	2.248	2.038	***
PHY	outdoor	2.836	2.675	***	2.857	2.751	***	2.642	2.547	**	2.642	2.383	***	2.816	2.540	***
	exercise	2.827	2.660	***	2.683	2.518	***	2.775	2.810	ns	2.958	2.776	***	3.227	3.048	***
	fit	3.157	2.956	***	3.108	2.857	***	2.959	2.942	ns	2.832	2.651	***	3.356	3.278	*
RES	private_pc	0.552	0.496	***	0.616	0.519	***	0.716	0.670	**	0.826	0.814	ns	0.357	0.402	*
	internet	0.855	0.855	ns	0.860	0.872	ns	0.904	0.913	ns	0.919	0.914	ns	0.810	0.789	ns
	quiet_space	0.826	0.830	ns	0.838	0.827	ns	0.755	0.798	**	0.689	0.693	ns	0.791	0.848	***
ICT	ict_gen	3.072	2.742	***	3.411	3.080	***	3.510	3.023	***	3.024	2.728	***	2.185	1.821	***
	ict_school	2.250	2.005	***	2.432	2.114	***	2.839	2.232	***	2.326	2.170	**	1.774	1.684	**

***Indicates significance at the 1% level, ** at the 5% level, * at the 10% level and ns: non-significant p-value. P-values refer to the Adjusted Wald test for equal means between boys and girls. Results are adjusted for survey setting with 75 Jackknife replications and 43 number of strata

**Fig. 2:**
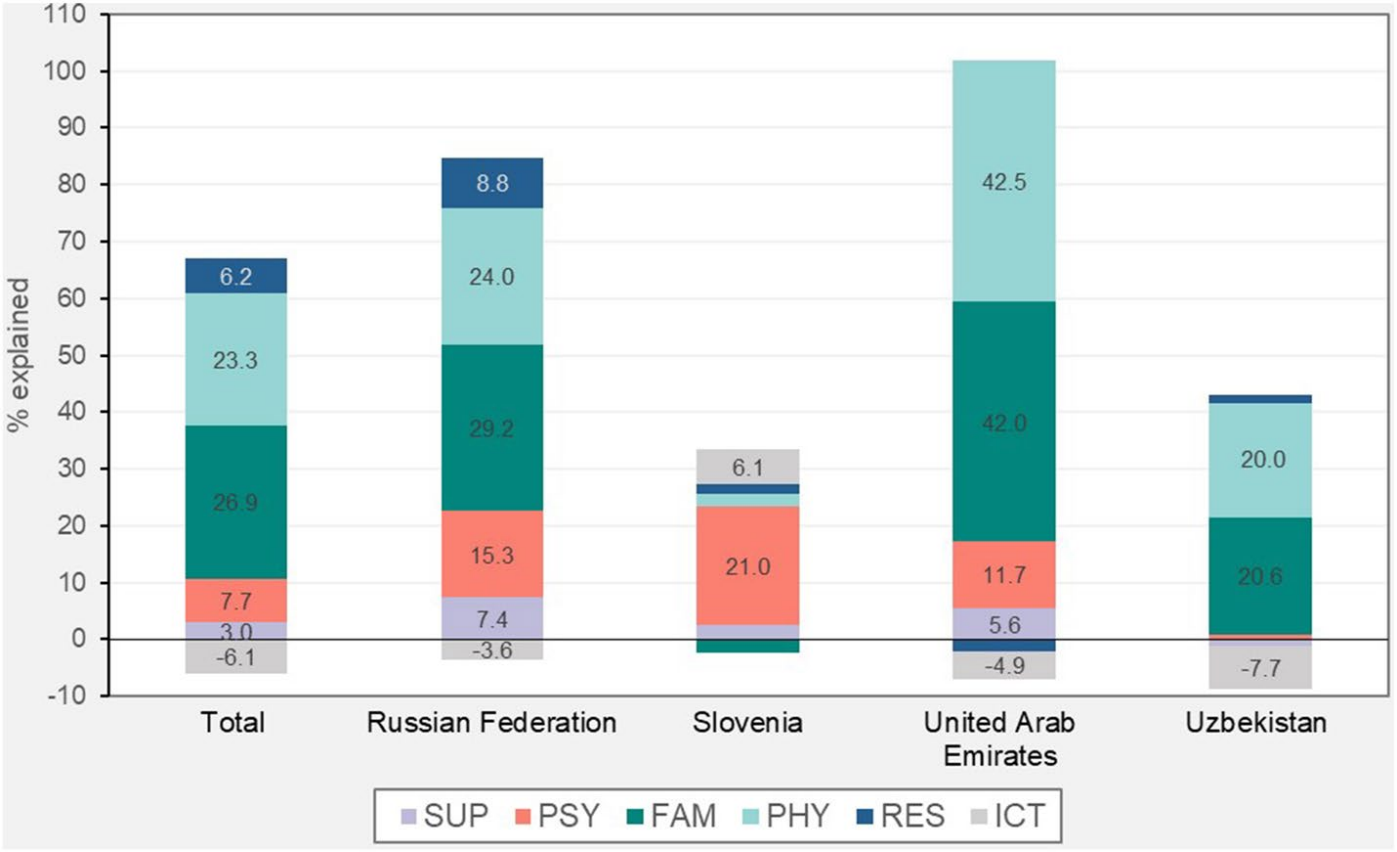
KHB decomposition of gender gap in improvement in perceived learning: details per channels. The figure reports the share of gender gap explained by each channel based on the KHB decomposition results displayed in Table 3. KHB decomposition per single variable is displayed in Table 10, in the Appendix. Labels with values below 3% are not reported to improve the graphic visualization

To examine how these differences affect the gender gap in students’ performance, a KHB decomposition for the two dependent variables is carried out.^11^ Table 5 reports the KHB estimates for students’ improvement in perceived learning. The results highlight that 60.93% of the difference between boys and girls in the total sample is accounted for by the proposed channels. The share of the explained gap is particularly large in the UAE (94.92%), while the lowest value is found for Slovenia (31.06%). Figure 2 displays the decomposition details of the gender gap in improvement in perceived learning for each channel (as described in Table 2). Except for Slovenia, family climate and physical activities seem to represent the most relevant factors (accounting respectively for 26.89% and 23.26% of the gender difference in the pooled model). The findings confirm the evidence available in some country-specific studies. In an analysis of secondary school students in the UAE, Bawa'aneh (2021) reveals that male students reported a better attitude toward studying at home, while females suffered more from the shift from traditional education to online learning in terms of family and home climate. Ermasova et al. (2022) show that male students in Russia are more likely to use physical exercise to cope with stress compared to females. In addition, psychological distress explains a large part of the gender gap in the Russian Federation, the UAE and, especially, in Slovenia, where this channel accounts for the greatest part of the observed difference between female and male students (20.98%). Household resources have, instead, a moderate relevance in explaining the gender gap in the Russian Federation (8.84%), but they are not influential in the other three countries analysed. Furthermore, support from family and teachers holds a marginal explanatory power in all the countries investigated. The same is for the ICT channel, but in the opposite direction (except for Slovenia). Indeed, despite girls reporting a lower level of digital skills before the pandemic, the ICT gap seems to slightly advantage female over male students in terms of learning outcomes. The interpretation of this finding is not straightforward but could be partially related to the collinearity with other channels. It could be the case that the positive correlation that the literature usually identifies between digital skills and academic performance during COVID-19 (Amaro et al., 2020; Di Pietro et al., 2020) is partially captured by the psychological distress of students. In other words, a lack of digital skills may indirectly influence the perceived learning by generating anxiety among girls that, in turn, affects their academic output. The proposed interpretation is supported by the estimates of the correlation matrix in Table 12, in the Appendix, which shows a negative correlation between digital skills (*ict_gen* and *ict_school*) and psychological distress—especially in terms of anxiety.

**Table 5:** KHB decomposition for improvement in perceived learning

Variables	Total sample	Russian Federation	Slovenia	United Arab Emirates	Uzbekistan
Reduced	− 0.290***	− 0.275***	− 0.364***	− 0.252***	− 0.329***
	(0.053)	(0.074)	(0.085)	(0.088)	(0.074)
Full	− 0.113**	− 0.052	− 0.251***	− 0.013	− 0.217***
	(0.054)	(0.077)	(0.091)	(0.088)	(0.077)
Difference	− 0.177***	− 0.223***	− 0.113**	− 0.240***	− 0.113***
	(0.023)	(0.035)	(0.046)	(0.046)	(0.029)
% Explained	60.930	81.120	31.060	94.920	34.210
Pseudo R2	0.050	0.060	0.070	0.080	0.030
Observations	10,348	3046	2166	2396	2740

***Indicates significance at the 1% level, ** at the 5% level and * at the 10% level. Standard errors in parentheses. Estimates with survey weights and including the following controls: age lang_istr, he_parents, ses, and country dummies

**Fig. 3:**
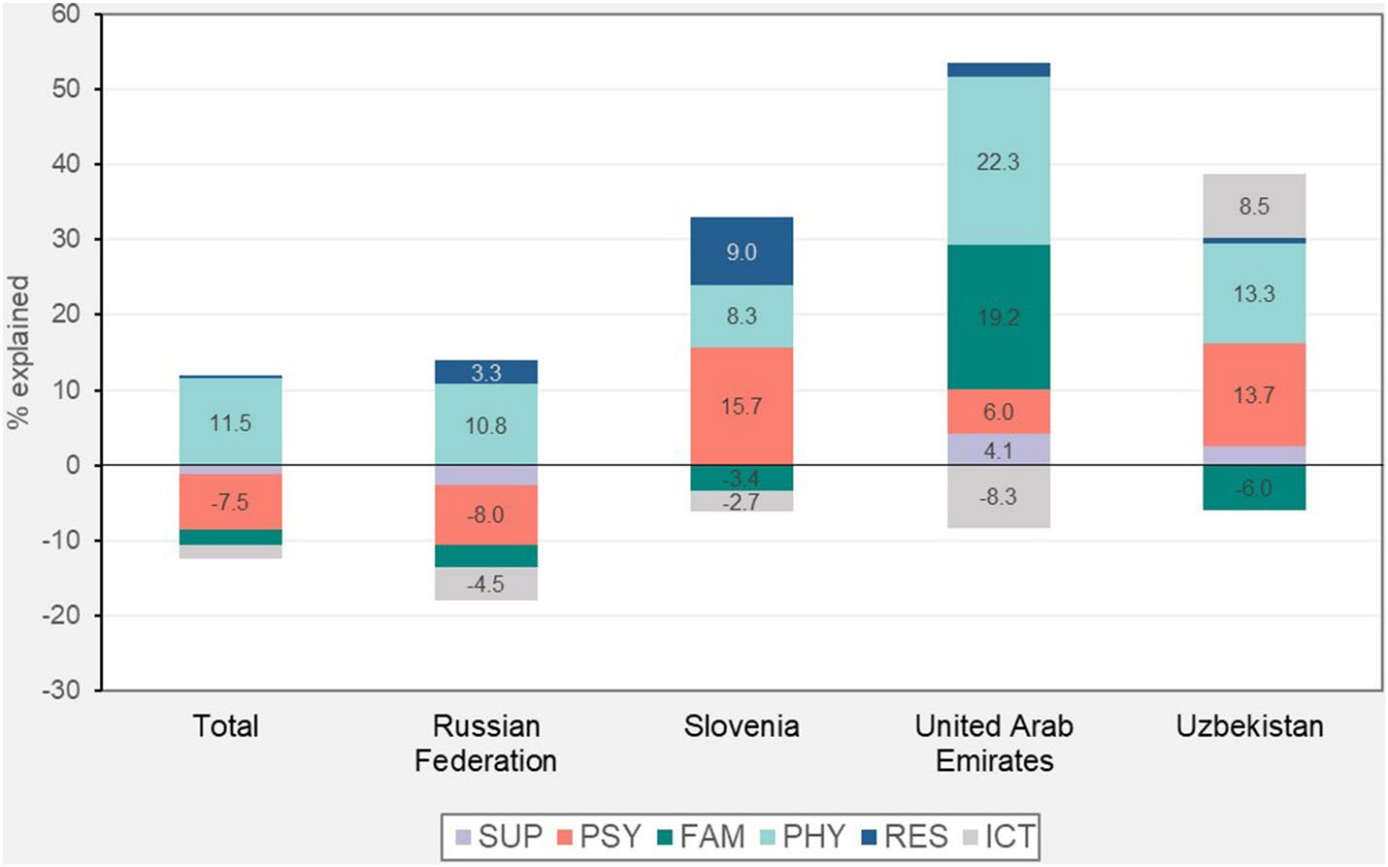
KHB decomposition of gender gap in improvement in grades: details per channels. The figure reports the share of gender gap explained by each channel based on the KHB decomposition results displayed in Table 4. KHB decomposition per single variable is displayed in Table 11, in the Appendix. Labels with values below 3% are not reported to improve the graphic visualization

Regarding the improvement in grades, the KHB decomposition reveals a not significant effect of the channel variables when considering the total sample, driven by a large heterogeneity across countries and channels (see Table 6 and Fig. 3). Overall, the channel variables account for a large share of the gender gap in the UAE (45.23%) and Uzbekistan (32.71%). However, the difference in the effect of gender between the full and the reduced model is not statistically significant in Slovenia and the Russian Federation. In line with the country-level results in Table 7, the coefficient on gender gap in Slovenia is indeed not statistically significant when improvement in grade is considered. This indicates that the disadvantage of girls due to the COVID-19 disruption in Slovenia is mainly related to the self-perceived evaluation of students and their expectations, but it is not evincible when a more objective measure is observed (i.e., differences in grades). To interpret this result, it should be considered the potential effect of teacher bias in the students grading in Slovenia. Indeed, Pavešić and Cankar (2019) find that, considering grade-8 students with similar standardised test scores in mathematics, girls tend to be graded with higher marks than boys. On the other hand, it is not possible to know if this behaviour has been amplified by the pandemic (having, thus, an effect on the dependent variable, which considers the difference between the pre and post COVID-19 period).^12^ In contrast, in the Russian Federation, the estimates confirm the presence of a significant gender gap that, however, cannot be explained by the proposed channels. This leaves space for alternative mechanisms that are not captured through the survey or that are too intrinsic for being measured by student self-reported data.

**Table 6:** KHB decomposition for improvement in grades

Variables	Total sample	Russian Federation	Slovenia	United Arab Emirates	Uzbekistan
Reduced	− 0.263***	− 0.309***	− 0.128	− 0.233***	− 0.151**
	(0.053)	(0.071)	(0.084)	(0.084)	(0.074)
Full	− 0.264***	− 0.321***	− 0.094	− 0.128	− 0.102
	(0.054)	(0.073)	(0.088)	(0.084)	(0.077)
Difference	0.001	0.012	− 0.034	− 0.105***	− 0.049*
	(0.014)	(0.022)	(0.037)	(0.028)	(0.026)
% Explained	− 0.460	− 3.900	26.730	45.230	32.710
Pseudo R2	0.030	0.010	0.020	0.030	0.020
Observations	10,348	3045	2162	2400	2741

***Indicates significance at the 1% level, ** at the 5% level and * at the 10% level. Standard errors in parentheses. Estimates with survey weights and including the following controls: age, lang_istr, he_parents, ses, and country dummies

In the UAE and Slovenia, the detailed decomposition by channel, reported in Fig. 3, is consistent with the results associated with the improvement in perceived learning. Physical activities and family climate account for most of the gender differences in the UAE (22.26% and 19.19%, respectively), while PHY is the main driver also in Uzbekistan. More generally, physical activity is confirmed as the most relevant channel, with consistent results across countries. Compared to the findings on improvement in perceived learning, family climate has, instead, a lower explanatory power. The only exception is the UAE, where this channel accounts for 19.19% of the observed differences between boys and girls. While the result for the UAE can be driven by country-specific characteristics, the overall findings suggest that the explanatory power of emotional and psychological factors is reduced when a more objective learning outcome is considered (i.e., student grades). This interpretation is supported by the results found for psychological distress, which has a lower influence compared to the results in Fig. 2, especially for the Russian Federation. As for improvement in perceived learning, SUP and RES channels are not particularly relevant, while ICT is associated with a mediation effect that goes in the opposite direction of the gender gap in the improvement in student grades. As discussed above, the result on the ICT channel can be due to the collinearity of these variables with psychological distress (see Table 12 in the Appendix).

## Conclusions

This study investigates gender gaps in learning performance as a consequence of the COVID-19 school disruption—and its potential drivers—using data from the REDS survey for four countries (i.e., the Russian Federation, Slovenia, Uzbekistan and the United Arab Emirates). To the best of the authors’ knowledge, this is the first study addressing such issue from a comparative perspective.

Pre-COVID-19 data show that, in three of the four countries here analysed, gender gaps in learning outcomes (as measured by standardised tests) existed prior to the pandemic. In general, boys tended to perform better than girls in mathematics, while the opposite was true in reading. However, these subject-specific gender gaps are not the focus of this study. The paper considers, indeed, whether boys and girls experienced different changes in (general) learning performances during physical school closure as a consequence of the COVID-19 pandemic. In other words, the analyses investigate whether the changes in learning performance from before to during/after the pandemic are gender-specific. Learning performance is measured using two indicators: (a) improvement in perceived learning (i.e., students’ self-evaluation of their academic performance during the lockdown period relative to the period before the lockdown); (b) improvement in grades (self-reported differences in student grades between the lockdown period and the period before the lockdown).

The first interesting result is that, for both indicators, boys perceived changes in their learning outcomes relative to the pre-COVID 19 period more favourably than girls: in the baseline model, the odds of an affirmative response are 24.47% (for improvement in perceived learning) and 21.69% (for improvement in grades) lower for girls. Such a gap is similar across the different countries, which are characterised by educational, cultural and socioeconomic heterogeneity. The extent to which such a result would hold for a larger set of countries and its persistence in time could only be addressed when new waves of international student assessment data (e.g., TIMSS, PIRLS and PISA) will become publicly available.

The negative coefficient on the dummy for female students in our regression models should not be interpreted as evidence of a gender gap in favour of boys in learning outcomes, especially when it comes to grades. While gaps in standardised test scores differ by subject, research has shown that girls receive consistently better grades than boys across all academic subjects (Enzi, 2015; Kiss, 2013; Lavy, 2008; Lievore and Triventi, 2022; Riegle-Crumb et al., 2018). A gender gap in grades favouring girls may continue to exist even if boys perceive their grades to have improved more than those of girls during the pandemic.

In order to better understand the potential drivers of the estimated gender gaps, the paper investigates the role of six potential channels that could account for them: psychological distress (PSY); support from teachers and family (SUP); family climate (FAM); physical activity and fitness (PHY); household resources for remote education (RES); ICT skills pre-COVID-19 (ICT).

First, the KHB decomposition analysis shows that the six channels, considered together, account for 60.93% of the observed gender gap in improvement in perceived learning. When considering improvement in grades, the channel variables seem to lose explanatory power: while in the UAE and Uzbekistan these variables still play a significant role (45.23% and 32.71%, respectively), they account for a low share of the gender gap in the Russian Federation. In this sense, the findings point to the fact that additional drivers—not included in this study due to unavailability of data—could play a relevant role in explaining differences in the changes in students’ grades between boys and girls.

Concerning the role of the different channels, family climate, physical activity/fitness and psychological distress are the main drivers of the gender gap in improvement in perceived learning during the pandemic. The ranking of these factors changes across countries but their relevance remains high in all countries (the only exception is Slovenia). Regarding the improvement in grades, PHY is confirmed as the main driver of the gender gap in all four countries (especially in the UAE). On the other hand, psychological distress and, in particular, family climate are less relevant to account for the gender gap observed for such dependent variable. This finding highlights the difference between the two dependent variables, discussed in Sect. “Data”. For its evaluative nature, self-perceived performance of students is likely to be affected by psychological and climate factors, whose influence is considerably attenuated when a more objective and factual measure of students’ achievement (such as grades) is considered.

Overall, the analysis confirms the relevance of emotional and psychological factors in impacting students’ learning—with a larger influence on girls compared to boys (see Pelch, 2018). REDS data, indeed, shows that female students reported higher psychological distress due to the COVID-19 school closure compared to boys, validating previous evidence in the literature (see, for instance, Mendolia et al., 2022; Prowse et al., 2021).

As for family climate, the literature shows that living in a safe and conflict-free environment exerts a positive effect on learning, especially in a remote learning environment like the one forced by the COVID-19 pandemic (see Pozzoli et al., 2022). Moreover, REDS data show that girls tend to score lower than boys in both the variables that comprise the FAM indicator. This could be the result of a higher sensitivity of girls with respect to the family climate, but it could also be related to actual different treatments of boys and girls (potentially exacerbated due to the pandemic and the lockdowns).

Students’ physical activity is found to explain a large part of the gender gap in terms of perceived improvement in both learning and grades and deserves, therefore, a closer look. On the one hand, several papers point to the existence of a positive correlation between students’ physical well-being and their learning outcomes (Donnelly et al., 2016)—especially during the lockdown, where outdoor activities were an effective coping strategy for young adults (Pigaiani et al., 2020). On the other hand, the results of this paper reveal that boys report higher values than girls in all the three variables that make up the PHY channel. The combination of these two factors could, by itself, explain the observed patterns. However, it is also possible that this channel captures other mechanisms associated to gender disparities (similar to those mentioned above for the FAM indicator). In particular, during school closure, girls could have seen a larger increase in domestic responsibilities compared to boys, with male students having more free time for outdoor activities than females. Based on this, the result may confirm the evidence in the literature that associates gender educational differences during the lockdown with an unequal division of domestic tasks between female and male children (see, for instance, Asanov et al., 2020).

Interestingly, but not unexpectedly, results indicate the pivotal role played by family climates during physical school closure. On the other hand, household resources for remote education and support from family and teachers seem to play a minor role.

Some limitations concern the two dependent variables. As discussed in Sect. “Data”, the data available from REDS survey are self-reported. This characteristic makes the indicators more prone to suffer from biases compared to more objective measures, such as standardised test scores. However, studies attempting to measure the accuracy of students’ self-assessments claim that there is some degree of correspondence between students’ self-assessments and external assessments, such as grades and test scores (Brown & Harris, 2013; Panadero et al., 2016). There also appears to be a consensus that students with longer experience in school tend to make more accurate judgements about their performance (Brown & Harris, 2013; Panadero et al., 2016). This is particularly important given that students participating in REDS were in grade 8 at the time of the survey and they had, thus, acquired some schooling experience already. As regards the second dependent variable, a number of studies have found self-reported grades to be a reasonably good proxy for actual grades (Cole & Goneya, 2010; Kuncel et al., 2005; Shaw & Mattern, 2009; Sticca et al., 2017)^13^. Importantly, no systematic differences in misreporting of grades by gender have been identified (Sticca et al., 2017).

In the next years, the publication of international large-scale assessment data (e.g., TIMSS, PIRLS and PISA) will allow a more precise estimate of the gender gap during the COVID-19 disruption. In the meanwhile, the dependent variables analysed in this paper can be considered as satisfactory proxies for the changes in students’ performance. Additionally, while students have been asked to make an overall assessment about how the pandemic affected their learning and grades, as pointed out at the end of Sect. “Gender differences in the effect of Covid-19 on students’ performance”, there may be relevant gender differences across subjects that could not be captured in our analysis. Finally, students have been asked to report differences in learning and grades between the COVID-19 and pre-COVID-19 periods. However, as shown by several studies (see, for example, van Gerwen et al., 2019), this could lead to reporting errors as respondents may recall more information about recent events than distant ones.

By reflecting on (some of) the determinants of the gender gap in students’ learning, this study offers information that could be used to design policy actions aimed at reducing the existing gender gap. Of the three main channels, schools can directly affect physical activities and, possibly, introduce measures to support students’ psychological well-being, while they cannot directly influence family climate. However, the role schools can play could be broader: they can provide complete and timely information to families on the learning goals and learning paths, as well as communicate regularly with the parents and inform them about the progress of their children. This would likely reduce the uncertainty about what is expected from students (and parents), with a positive effect on students’ (and parents’) anxiety and on family climate.

It is evident that schools and teachers were not ready to provide collective and individualised support to families and students, also because they had to adjust to the shift to online learning (and the stress of the lockdown). As it appears that the negative effects of the pandemic are now more under control (mainly thanks to the vaccines), policy makers, schools, teachers, families and students should devote some time to identify the best ways in which to communicate and support each other. This will increase the resilience of education systems to external shocks (such as the COVID-19 emergency) and rise the perception of the fundamental role played by all the stakeholders in the creation of the human capital of the future.

## Figures and Tables

**Table 12:**
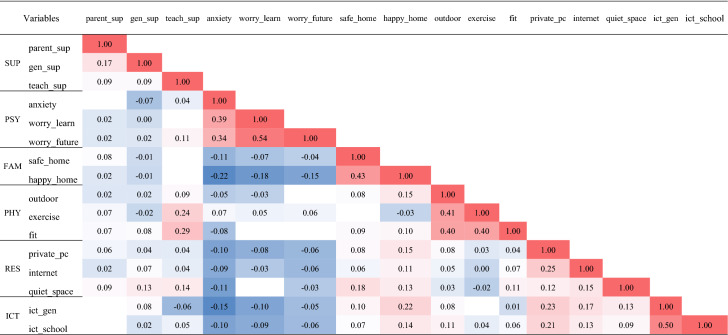
Correlation matrix of channel variables

Pairwise correlation coefficients. Only the statistically significant values (p < 0.05) are displayed. Survey weights are used

## Data Availability

The datasets analysed during the current study are available in the Responses to Educational Disruption Survey (REDS) repository, https://www.iea.nl/index.php/data-tools/repository/reds.

## Appendix

See Tables 7, 8, 9, 10, 11, 12.

**Table 7:** Ordered logit on improvement in perceived learning and improvement in grades by country, odds ratios estimates

Variables	Russian Federation		United Arab Emirates		Slovenia		Uzbekistan	
	IPL	IG	IPL	IG	IPL	IG	IPL	IG
female	0.762***	0.752***	0.832*	0.810***	0.729***	0.868*	0.729***	0.874*
	(0.065)	(0.050)	(0.078)	(0.060)	(0.067)	(0.068)	(0.060)	(0.064)
age	1.069	1.023	1.123**	1.101	0.999	0.859	1.220**	0.974
	(0.010)	(0.059)	(0.061)	(0.066)	(0.123)	(0.102)	(0.110)	(0.090)
ses	1.006	0.998	0.978***	0.991*	0.999	1.001	1.002	0.988***
	(0.005)	(0.005)	(0.005)	(0.005)	(0.004)	(0.005)	(0.005)	(0.005)
lang_istr	0.999	0.775*	1.035	0.909	0.970	1.185	1.313*	1.112
	(0.135)	(0.101)	(0.098)	(0.089)	(0.165)	(0.164)	(0.191)	(0.154)
he_parents	0.971	1.039	1.187*	1.081	1.189*	0.923	0.952	1.037
	(0.080)	(0.098)	(0.109)	(0.121)	(0.113)	(0.087)	(0.085)	(0.094)
cut1	0.874	0.154*	0.562	0.380	0.303	0.0164**	5.107	0.0288**
	(1.151)	(0.142)	(0.475)	(0.314)	(0.534)	(0.028)	(6.419)	(0.040)
cut2	9.385	1.073	2.818	1.915	2.353	0.010	28.890***	0.160
	(12.23)	(0.987)	(2.372)	(1.589)	(4.141)	(0.169)	(36.080)	(0.217)
cut3	38.040**	4.521	12.060***	9.710***	10.270	0.535	115.3***	0.952
	(50.310)	(4.155)	(10.440)	(8.143)	(18.130)	(0.907)	(145.300)	(1.299)
Pseudo R2	0.002	0.003	0.006	0.004	0.004	0.001	0.005	0.002
Observations	3411	3399	2695	2685	2388	2381	2878	2873

***Indicates significance at the 1% level, ** at the 5% level and * at the 10% level. IPL indicates the dependent variable improvement in perceived learning, while IG the improvement in grades. Coefficients are reported in odds ratios. Jackknife standard errors in parentheses. Results are adjusted for survey setting with 75 Jackknife replications. Pseudo R2 is not provided for survey setting. The values of Pseudo R2 are taken from simple ordered logistic models, without survey parameters

**Table 8:** Ordered logit on improvement in perceived learning including channel variables: country details, odds ratios estimates

Variables	(1)	(2)	(3)	(4)
	Russian Federation	Slovenia	United Arab Emirates	Uzbekistan
female	0.963	0.794**	1.009	0.807**
	(0.075)	(0.079)	(0.107)	(0.070)
parent_sup	0.911	1.012	0.760***	1.058
	(0.098)	(0.091)	(0.076)	(0.079)
gen_sup	0.964	0.852	0.938	0.723***
	(0.089)	(0.093)	(0.087)	(0.073)
teach_sup	1.385***	1.109	1.423***	1.057
	(0.104)	(0.096)	(0.151)	(0.087)
anxiety	0.926	0.797***	0.793***	1.056
	(0.055)	(0.052)	(0.050)	(0.055)
worry_learn	0.798***	0.945	0.802***	0.917
	(0.055)	(0.075)	(0.053)	(0.054)
worry_future	0.966	0.792***	1.007	1.000
	(0.064)	(0.054)	(0.068)	(0.056)
safe_home	1.172***	1.257***	1.362***	1.157***
	(0.046)	(0.052)	(0.063)	(0.046)
happy_home	1.464***	1.365***	1.414***	1.267***
	(0.071)	(0.073)	(0.087)	(0.052)
outdoor	1.045	1.136**	1.211***	1.127**
	(0.049)	(0.060)	(0.078)	(0.059)
exercise	1.197**	1.258***	1.061	1.102
	(0.082)	(0.081)	(0.064)	(0.083)
fit	1.140**	1.075	1.281***	1.180*
	(0.067)	(0.078)	(0.108)	(0.101)
private_pc	1.307**	1.200*	1.242*	1.341**
	(0.125)	(0.123)	(0.155)	(0.148)
internet	1.043	1.102	0.706**	1.099
	(0.156)	(0.170)	(0.112)	(0.107)
quiet_space	0.794*	1.042	0.849	0.822*
	(0.095)	(0.129)	(0.103)	(0.090)
ict_gen	0.935	0.959	0.965	0.942
	(0.039)	(0.048)	(0.036)	(0.042)
ict_school	1.045	1.059*	0.971	0.986
	(0.042)	(0.034)	(0.037)	(0.046)
Controls	Yes	Yes	Yes	Yes
Country FE	No	No	No	No
cut1	3.900	0.377	5.047*	14.422**
	(5.744)	(0.759)	(4.851)	(18.330)
cut2	55.032**	3.792	34.132***	92.078***
	(79.753)	(7.613)	(33.171)	(117.457)
cut3	249.940***	19.630	178.250***	389.810***
	(366.034)	(39.393)	(176.397)	(504.598)
Pseudo R2	0.061	0.067	0.086	0.030
Observations	3046	2166	2396	2740

***Indicates significance at the 1% level, ** at the 5% level and * at the 10% level. Coefficients are reported in odds ratios. Jackknife standard errors in parentheses. All models include the following controls: age, ses, he_parents and lang_istr. Results are adjusted for survey setting with 75 Jackknife replications and 43 number of strata. Pseudo R2 is not provided for survey setting. The values of Pseudo R2 are taken from simple ordered logistic models, without survey parameters

**Table 9:** Ordered logit on improvement in grades including channel variables: country details, odds ratios estimates

Variables	(1)	(2)	(3)	(4)
	Russian Federation	Slovenia	United Arab Emirates	Uzbekistan
female	0.726***	0.910	0.880*	0.903
	(0.054)	(0.076)	(0.067)	(0.067)
parent_sup	0.956	1.112	1.012	0.945
	(0.083)	(0.099)	(0.100)	(0.084)
gen_sup	1.294**	0.964	1.055	0.819**
	(0.114)	(0.078)	(0.096)	(0.062)
teach_sup	0.928	1.041	1.389***	0.974
	(0.076)	(0.110)	(0.111)	(0.0771)
anxiety	1.114	0.932	0.766***	1.374**
	(0.089)	(0.090)	(0.075)	(0.167)
worry_learn	1.118	0.862	1.060	1.226*
	(0.123)	(0.082)	(0.141)	(0.127)
worry_future	1.135	1.011	1.068	1.516***
	(0.110)	(0.099)	(0.149)	(0.159)
safe_home	1.038	1.142***	1.124**	0.983
	(0.044)	(0.051)	(0.052)	(0.033)
happy_home	0.927*	1.233***	1.178***	0.968
	(0.039)	(0.059)	(0.052)	(0.037)
outdoor	1.004	1.150**	1.093	1.066
	(0.052)	(0.072)	(0.068)	(0.059)
exercise	1.079	1.124**	1.031	1.003
	(0.057)	(0.062)	(0.083)	(0.057)
fit	1.085	1.032	1.131*	1.012
	(0.064)	(0.076)	(0.083)	(0.070)
private_pc	1.097	1.329***	1.004	0.832*
	(0.067)	(0.129)	(0.136)	(0.088)
internet	0.857	0.892	1.150	0.814*
	(0.102)	(0.150)	(0.264)	(0.086)
quiet_space	0.943	1.086	1.100	0.912
	(0.105)	(0.110)	(0.139)	(0.094)
ict_gen	0.996	1.013	0.938*	1.028
	(0.042)	(0.053)	(0.034)	(0.039)
ict_school	0.960	0.984	0.985	1.017
	(0.033)	(0.039)	(0.032)	(0.043)
Controls	Yes	Yes	Yes	Yes
Country FE	No	No	No	No
cut1	0.253	0.034*	5.110*	0.063**
	(0.281)	(0.071)	(4.715)	(0.083)
cut2	1.784	0.228	27.710***	0.362
	(1.979)	(0.450)	(25.83)	(0.468)
cut3	7.821*	1.293	154.100***	2.262
	(8.611)	(2.556)	(144.900)	(2.926)
Pseudo R2	0.007	0.023	0.028	0.016
Observations	3045	2162	2400	2741

***Indicates significance at the 1% level, ** at the 5% level and * at the 10% level. Coefficients are reported in odds ratios. Jackknife standard errors in parentheses. All models include the following controls: age, ses, he_parents and lang_istr. Results are adjusted for survey setting with 75 Jackknife replications and 43 number of strata. Pseudo R2 is not provided for survey setting. The values of Pseudo R2 are taken from simple ordered logistic models, without survey parameters

**Table 10:** Share of explained gender gap in improvement in perceived learning: details per channels and variables

Variables	Total	Russian Federation	Slovenia	United Arab Emirates	Uzbekistan
SUP	3.0	7.4	2.5	5.6	− 1.0
PSY	7.7	15.3	21.0	11.7	0.9
FAM	26.9	29.2	− 2.4	42.0	20.6
PHY	23.3	24.0	2.0	42.5	20.0
RES	6.2	8.8	1.8	− 2.0	1.4
ICT	− 6.1	− 3.6	6.1	− 4.9	− 7.7
*Details per single variable*					
parent_sup	− 0.1	− 0.7	0.0	− 0.4	− 0.4
gen_sup	0.4	0.1	2.3	1.1	0.3
teach_sup	2.7	8.0	0.2	4.9	− 0.9
anxiety	1.5	4.4	4.6	9.0	0.7
worry_learn	4.5	6.8	4.2	4.1	0.4
worry_future	1.8	4.1	12.2	− 1.4	− 0.2
safe_home	5.8	5.8	− 8.3	11.1	5.2
happy_home	21.1	23.5	5.9	30.9	15.4
outdoor	4.9	2.0	3.2	18.6	10.4
exercise	9.2	11.0	− 2.0	3.9	5.5
fit	9.2	11.0	0.8	20.0	4.1
private_pc	6.1	10.3	2.4	1.2	− 2.1
internet	0.1	− 0.1	0.0	− 1.9	1.0
quiet_space	0.0	− 1.4	− 0.6	− 1.3	2.5
ict_gen	− 8.3	− 8.6	− 4.8	− 3.5	− 7.1
ict_school	2.2	5.0	10.9	− 1.4	− 0.5

The table reports the share of gender gap explained by each channel and variable based on the KHB decomposition results displayed in Table 5. Stata 15, the software used for the empirical analyses, reports only one decimal value in the output

**Table 11:** Share of explained gender gap in improvement in grades: details per channels and variables

Variables	Total	Russian Federation	Slovenia	United Arab Emirates	Uzbekistan
SUP	− 1.1	− 2.6	− 0.1	4.1	2.5
PSY	− 7.5	− 8.0	15.7	6.0	13.7
FAM	− 2.0	− 2.9	− 3.4	19.2	− 6.0
PHY	11.5	10.8	8.3	22.3	13.3
RES	0.4	3.3	9.0	2.0	0.8
ICT	− 1.7	− 4.5	− 2.7	− 8.3	8.5
*Details per single variable*					
parent_sup	− 0.1	− 0.3	− 2.2	0.0	0.9
gen_sup	− 0.4	− 0.6	1.7	− 1.2	0.4
teach_sup	− 0.6	− 1.7	0.4	5.3	1.2
anxiety	− 2.2	− 2.4	3.4	7.5	7.9
worry_learn	− 2.1	− 2.4	13.4	− 0.6	− 1.1
worry_future	− 3.3	− 3.2	− 1.1	− 0.8	6.9
safe_home	0.8	1.2	− 13.9	4.5	− 1.4
happy_home	− 2.9	− 4.1	10.5	14.7	− 4.6
outdoor	1.9	0.2	10.3	9.4	12.2
exercise	3.5	4.1	− 3.0	2.6	0.4
fit	6.2	6.6	1.0	10.3	0.7
private_pc	0.6	3.2	11.2	0.0	2.9
internet	− 0.2	0.4	0.0	0.9	− 4.7
quiet_space	0.0	− 0.3	− 2.3	1.0	2.5
ict_gen	0.3	− 0.4	5.1	− 7.4	7.1
ict_school	− 2.0	− 4.1	− 7.8	− 0.9	1.4

The table reports the share of gender gap explained by each channel and variable based on the KHB decomposition results displayed in Table 6

## Abbreviations

FAM: Family climate (channel)
FE: Fixed effects
GII: Gender Inequality Index
ICT: ICT skills pre-COVID-19 (channel)
IG: Improvement in grades
IPL: Improvement in perceived learning
KHB: Karlson-Holm-Breen
PHY: Physical activity and fitness (channel)
PSY: Psychological distress (channel)
REDS: Responses to Educational Disruption Survey
RES: Household resources for remote education (channel)
SUP: Support from teachers and family (channel)
UAE: United Arab Emirates

## Footnotes

1 Lockdown period is defined as the period in which most schools in a country were closed for the majority of the students. Thus, this time span varies across countries and schools but is generally distributed between March and June 2020 (Meinck et al., 2022).

2 REDS student data are also available for Burkina Faso, Denmark, Ethiopia and Kenya. However, these countries may not be representative of the target population and present a large number of non-respondents and missing data, including replicate weights (Meinck et al., 2022). For these reasons, the analyses presented here focus on four countries: the Russian Federation, Slovenia, the UAE and Uzbekistan.

3 Additional information on how the four countries responded to the COVID-19 disruption and detailed information on their educational systems are available in Meinck et al. (2022).

4 Given the traditional importance of parental education, the models include a dummy indicating if at least one of the parents has achieved higher education attainment separately from student socioeconomic status (see Pokropek et al., 2015). This empirical approach is frequent in the literature (see, for instance, Masci et al., 2018). Also, the results without parents’ higher education variables (available upon request) do not show significant changes in the estimates.

5 Domestic responsibilities have not been included within the channels for two reasons. First, the information gathered through the survey can only partially capture these activities. REDS survey provides information on caring for siblings and older relatives, but no data are available on other domestic responsibilities (cooking, cleaning, etc.). Second, it was not possible to control for the family composition in terms of the gender of the siblings, which could cause biased results. However, additional analyses that include variables on siblings and elderly care are available upon request.

6 ${\beta }_{R}$ and ${\beta }_{F}$ are the coefficients of the latent variable model associated with the ordered logit model.

7 The interactions between country fixed effects and gender are not statistically significant when included in the baseline models (i.e., Columns 1 and 3). The analyses are available upon request to the authors.

8 The GII is a composite indicator measuring gender inequality based on three dimensions: reproductive health (maternal mortality ratio and adolescent birth rate), empowerment (share of parliamentary seats held by women and share of population with at least some secondary education) and the labour market (labour force participation rate). Low values of GII are associated with low inequality between women and men (and vice-versa) (UNDP, 2022).

9 In 2019, the GII value for Slovenia was 0.063, while the UAE reported a value of 0.079, the Russian Federation a value of 0.225, and Uzbekistan a value of 0.288 (UNDP, 2022).

10 Such finding is consistent with evidence from Slovenia showing that female students have lower levels of digital skills than their male counterparts (European Commission, 2020).

11 The share of gender difference explained by each channel is obtained by summing the contributions of the variables composing the channels, which are reported in Tables 10 and 11 of the Appendix. Similar results are found by performing the KHB decomposition with aggregated indexes of the six channels (estimates available upon request).

12 Based on the REDS teacher survey, it is only possible to say that 60% of the teachers reported to have used the same grading criteria as before the pandemic.

13 While improvement in grades is also based on students’ self-reports, this indicator is factual in nature in the sense that students’ answers are, at least in principle, verifiable: had the grades of the students participating in REDS been made available to us, we could verify how accurate students’ assessments of improvement in grades are.
